# Construction of Marker-Free Transgenic Strains of *Chlamydomonas reinhardtii* Using a Cre/*loxP*-Mediated Recombinase System

**DOI:** 10.1371/journal.pone.0161733

**Published:** 2016-08-26

**Authors:** Yuki Kasai, Shigeaki Harayama

**Affiliations:** Department of Biological Sciences, Faculty of Science and Engineering, Chuo University, Bunkyo-ku, Tokyo, Japan; University of Texas at Austin Dell Medical School, UNITED STATES

## Abstract

The *Escherichia coli* bacteriophage P1 encodes a site-specific recombinase called Cre and two 34-bp target sites of Cre recombinase called *loxP*. The Cre/*loxP* system has been used to achieve targeted insertion and precise deletion in many animal and plant genomes. The Cre/*loxP* system has particularly been used for the removal of selectable marker genes to create marker-free transgenic organisms. For the first time, we applied the Cre/*loxP*-mediated site-specific recombination system to *Chlamydomonas reinhardtii* to construct marker-free transgenic strains. Specifically, *C*. *reinhardtii* strains cc4350 and cc124 carrying an *aphVIII* expression cassette flanked by two direct repeats of *loxP* were constructed. Separately, a synthetic Cre recombinase gene (*CrCRE*), the codons of which were optimized for expression in *C*. *reinhardtii*, was synthesized, and a *CrCRE* expression cassette was introduced into strain cc4350 carrying a single copy of the *loxP*-flanked *aphVIII* expression cassette. Among 46 transformants carrying the *CrCRE* expression cassette stably, the excision of *aphVIII* by CrCre recombinase was observed only in one transformant. We then constructed an expression cassette of an in-frame fusion of *ble* to *CrCRE* via a short linker peptide. The product of *ble* (Ble) is a bleomycin-binding protein that confers resistance to bleomycin-related antibiotics such as Zeocin and localizes in the nucleus. Therefore, the *ble-*(linker)-*CrCRE* fusion protein is expected to localize in the nucleus. When the *ble-*(linker)-*CrCRE* expression cassette was integrated into the genome of strain cc4350 carrying a single copy of the *loxP*-flanked *aphVIII* expression cassette, CrCre recombinase-mediated excision of the *aphVIII* expression cassette was observed at a frequency higher than that in stable transformants of the *CrCRE* expression cassette. Similarly, from strain cc124 carrying a single *loxP*-flanked *aphVIII* expression cassette, the *aphVIII* expression cassette was successfully excised after introduction of the *ble-*(linker)-*CrCRE* expression cassette. The *ble-*(linker)-*CrCRE* expression cassette remained in the genome after excision of the *aphVIII* expression cassette, and it was subsequently removed by crossing with the wild-type strain. This precise Cre-mediated deletion method applicable to transgenic *C*. *reinhardtii* could further increase the potential of this organism for use in basic and applied research.

## Introduction

The green unicellular alga *Chlamydomonas reinhardtii* has been widely used as a model system for studying the genetic and molecular mechanisms of biological processes such as photosynthesis and flagellar motility [1, 2, 3]. Recently, this alga has also been used to manipulate metabolic pathways involved in biofuel and hydrogen production using the range of genetic manipulation tools available to this organism [4, 5, 6, 7]. However, the number of selectable marker genes used in *C*. *reinhardtii* is limited even though availability of multiple selectable markers is necessary for the sequential introduction of transgenes.

*C*. *reinhardtii* is considered to be a model organism for basic research and an industrial biotechnology host [8]. For the large-scale deployment of transgenic *C*. *reinhardtii* for various industrial applications, there are public concerns regarding the spread of marker genes in the environment.

Therefore, efficient methods for the removal of marker genes from transgenic *C*. *reinhardtii* are highly anticipated. Sexual crossing is a powerful tool for this purpose. However, this technique cannot be used if the linkage between a marker gene and a co-introduced transgene is tight. Such tight linkage between a marker gene and co-introduced transgenes was observed in transgenic rice and soybean generated by biolistic bombardment, in which most of the transgenes were co-integrated together with a marker gene at one or multiple loci [9, 10, 11]. Co-transformation of plants by *Agrobacterium tumefaciens*-mediated transformation using multiple plasmids also resulted in the integration of multiple T-DNAs at the same locus on plant chromosomes [12, 13, 14]. Thus, although the fate of multiple-plasmid co-transformation in *C*. *reinhardtii* was not examined systematically, we presume that multiple plasmids are frequently integrated at the same locus, leading to a tight genetic linkage between marker genes and co-introduced transgenes in transgenic *C*. *reinhardtii*.

One strategy to increase co-transformation frequency is the use of a marker gene physically linked to a gene of interest [15]. Several vectors systems were developed for this purpose [16, 17], including those enabling sustained expression of transgenes in recipients [18]. In cases in which transgenes were obtained using such vectors, the marker gene and transgene are genetically linked and usually inherited together.

The genomic sequence of *C*. *reinhardtii* includes numerous functionally uncharacterized genes [19]. Reverse genetics is a robust method for revealing the functions of such genes. Because *C*. *reinhardtii* displays an extremely low efficiency of homologous recombination [20, 21, 22], insertional random mutagenesis using selectable markers in *C*. *reinhardtii* was identified as a valuable tool for investigating diverse biological functions [23, 24, 25, 26, 27, 28, 29]. Although the removal of selectable markers from insertional mutants without the loss of mutant phenotypes is desired for further genetic manipulation or industrial application, marker rescue from insertional mutants using sexual crossing is not possible.

To overcome the limitations of sexual crossing, several strategies have been developed to remove selectable markers from transgenic eukaryotic cells [30], including the use of site-specific DNA excision systems such as Cre/*loxP* from bacteriophage P1 [31, 32, 33, 34], Flp/frt from *Saccharomyces cerevisiae* [35, 36], R/RS from *Zygosaccharomyces rouxii* [37, 38], and Gin/gix from bacteriophage Mu [39]. In the bacteriophage P1 bipartite Cre/*loxP*-mediated site-specific DNA excision system, Cre recombinase specifically recognizes the *loxP* sequence of 34 bp in length and excises a DNA segment flanked by two direct repeats of *loxP*, leaving a single copy of *loxP* [40, 41, 42]. This system has been proven to be a powerful marker rescue tool in eukaryotes [43, 44, 45].

Curiously, no research on the use of Cre/*loxP*-mediated system in *C*. *reinhardtii* has been published. In this study, we discuss the exact excision of a marker gene from the nuclear genome of *C*. *reinhardtii* via Cre/*loxP*-mediated site-specific recombination. This report expands the list of available genetics tools in this organism.

## Materials and Methods

### Plasmid Construction

**Fig 1:**
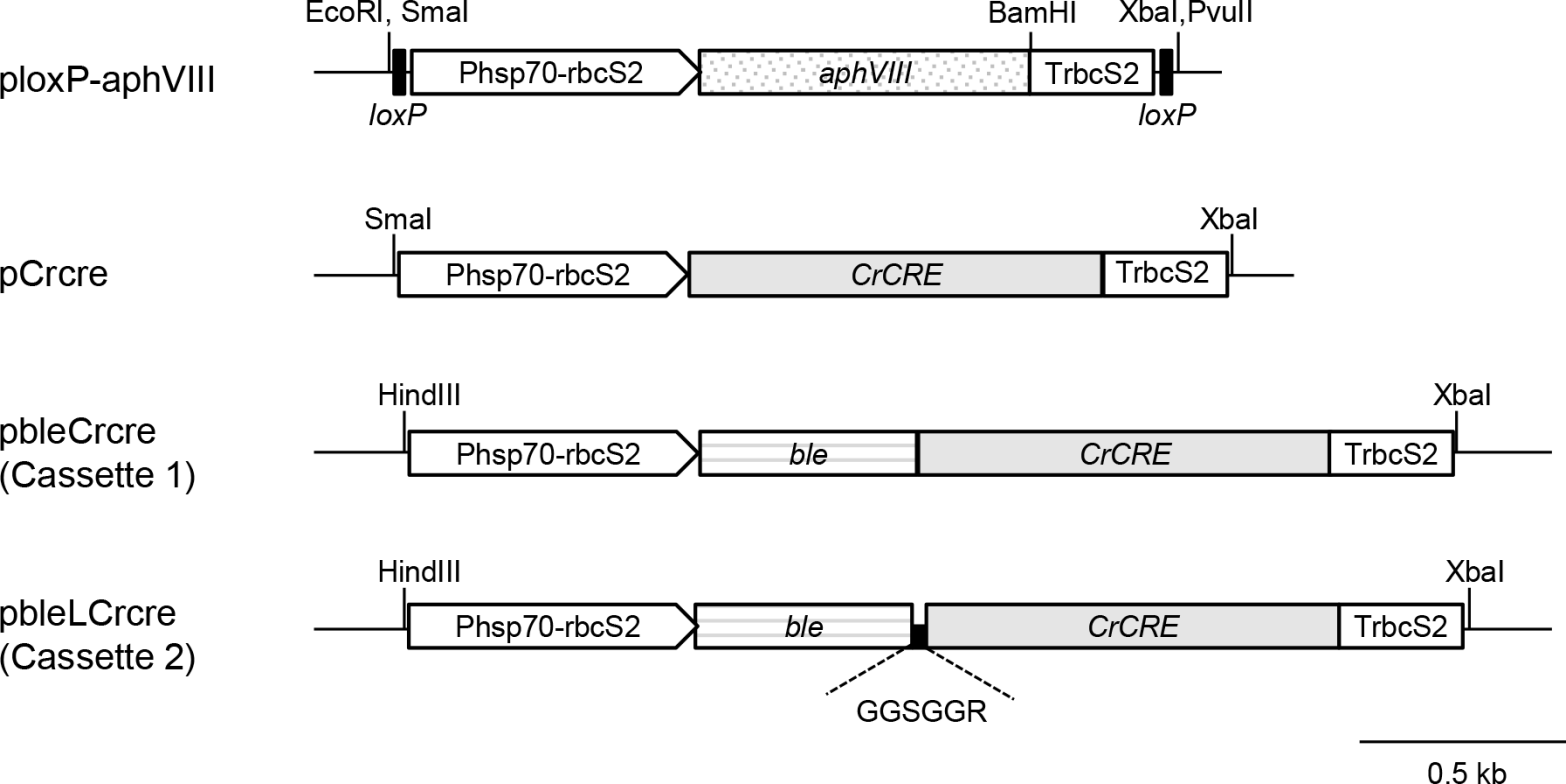
Structures of plasmids used in this study. The abbreviations of genes and loci were as follows: *aphVIII*, the gene for aminoglycoside 3′-phosphotransferase type VIII conferring paromomycin resistance; *CrCRE*, the codon-optimized gene for Cre recombinase; *ble*, the gene for bleomycin/Zeocin-binding protein conferring bleomycin/Zeocin resistance; Phsp70-rbcS2, the artificial tandem promoter consisting of the *HSP70A* and *RBCS2* promoters; TrbcS2, the terminator of *RBCS2*.

PCR for plasmid construction was performed using PrimeSTAR Max DNA polymerase (Takara) and appropriate primers, the sequences of which are listed in Table 1. *aphVIII* from *Streptomyces rimosus* encodes aminoglycoside 3′-phosphotransferase type VIII and confers resistance to paromomycin. The pSI103 plasmid carries the *aphVIII* expression cassette consisting of the *C*. *reinhardtii HSP70*_*RBCS2* promoter, *aphVIII*, and the *RBCS2* terminator [46]. For PCR amplification of the *aphVIII* expression cassette flanked by two direct repeats of *loxP* (*loxP-P-aphVIII-T-loxP*), the pSI103 plasmid was used as a template, and loxPphsp70_F1 and loxPtrbcS_R1 were employed as primers. The amplified fragment was digested using *Sma*I and *Xba*I and inserted between the *Sma*I and *Xba*I sites of the pBluescript II SK (+) plasmid to construct the ploxP-aphVIII plasmid (Fig 1).

**Table 1:** Primers used in this study.

Primer	Sequence (5′ to 3′)	Underlined sequence
Primers for plasmid construction		
phsp70_F1	TCCCCGGGATAACTTCGTATAGCATACATTATACGAAGTTATGAGCTCGCTGAGGCTTGACA	SmaI site
trbcS_R1	CCTCTAGAATAACTTCGTATAATGTATGCTATACGAAGTTATCGCTTCAAATACGCCCAGCC	XbaI site
phsp70_F2	TCCCCGGGGAGCTCGCTGAGGCTTGACA	SmaI site
prbcS_R1	TCAGCAGGTTGCTCATGCTTCGAAATTCTTCAGCACCGG	Underlined sequence is complementary to the underlined sequence of Crcre_F1
trbcS_F1	CGCCTGCTGGAGGACGGCGACTAAGGATCCCCGCTCCG	Underlined sequence is complementary to the underlined sequence of Crcre_R1
trbcS_R2	CACTCTAGAGCTTCAAATACGCCCAGCCC	XbaI site
Crcre_F1	GAAGAATTTCGAAGCATGAGCAACCTGCTGACCGTGCACC	Underlined sequence is complementary to the underlined sequence of prbcS_R1
Crcre_R1	CGGAGCGGGGATCCTTAGTCGCCGTCCTCCAGCAGGCG	Underlined sequence is complementary to the underlined sequence of trbcS_F1
phsp70_F3	CACAAGCTTGACGGCGGGGAGCTCGCTGA	HindIII site
ble_R	TTCTGGTGCACGGTCAGCAGGTTGTCCTGCTCCTCGGCCACG	Underlined sequence is complementary to the underlined sequence of Crcre_F2
Crcre_F2	GCCGAGGAGCAGGACAACCTGCTGACCGTGCACCAGAAC	Underlined sequence is complementary to the underlined sequence of ble_R
ble_R2	GCGGCCGCCGGAGCCGCCGTCCTGCTCCTCGGCCACGAAGTG	Underlined sequence encodes a linker peptide, and is complementary to the underlined sequence of Crcre_F3
Crcre_F3	GGCGGCTCCGGCGGCCGCATGAGCAACCTGCTGACCGTGCACCA	Underlined sequence encodes a linker peptide, and is complementary to the underlined sequence of ble_R2
Primers used for the detection of specific sequences		
aphVIII_F	ATGGACGATGCGTTGCGT	
aphVIII_R	TCAGAAGAACTCGTCCAAC	
loxP_F	AGCCCGGGATAACTTCGTA	
loxP_R	GGCCGCTCTAGAATAACTTCGT	
Crcre_F4	GAGCACACCTGGAAGATGCT	
Crcre_R2	CAGGTAGTTGTTGGGGTCGT	
trbcS_inv_F1	GCGGTGGATGGAAGATACTGCTCTC	
aphVIII_F2	CGACTTGGAGGATCTGGACG	
phsp70_inv_R2	CCGCCAAATCAGTCCTGTAGCTTCA	
trbcS_inv_F2	AGTTTTGCAATTTTGTTGGTTGT	
trbcS_inv_R	GGGGCAAGGCTCAGATCAAC	
LPm1_F2	TCTGATTTTGACTGATTTCGAGGC	
LPm1_R4	GGACAGGTATCCGGTAAGCG	
LPm19_F	AGCACCGTGCACCACCTGCCTGCGCA	
LPm19_R	GCGTTGGCCGATTCATTAATGCAGCT	

The codons of the Cre recombinase gene were optimized on the basis of the nuclear codon usage of *C*. *reinhardtii* stored in the codon usage database at Kazusa DNA Research Institute (http://www.kazusa.or.jp/codon/). Codon optimization was performed using the OptimumGene™ algorithm, and the optimized gene (*CrCRE*) was synthesized by GenScript (New Jersey, USA). The *CrCRE* sequence was cloned into the pUC57 plasmid to create the pUCrcre plasmid. The pCrcre plasmid (Fig 1) carrying the *CrCRE* sequence flanked by the *HSP70*-*RBCS2* promoter and *RBCS2* terminator (hereafter referred as “the *CrCRE* expression cassette”) was constructed using an overlapping PCR method as follows. In the first step, three DNA fragments were amplified separately using PCR: the 0.7-kb *HSP70*-*RBCS2* promoter sequence was amplified using phsp70_F2 and prbcS_R1 as primers and the pSI103 plasmid as a template; the 0.3-kb *RBCS2* terminator sequence was amplified using trbcS_F1 and trbcS_R2 as primers and the pSI103 plasmid as a template; and the 1.0-kb *CrCRE* sequence was amplified using Crcre_F1 and Crcre_R1 as primers and the pUCrcre plasmid as a template. In the second step, the three fragments amplified in the first step were assembled into a single fragment by PCR using the three fragments as templates and phsp70_F2 and trbcS_R2 as primers. The amplified product was purified using a PCR purification kit (Qiagen), digested with *Sma*I and *Xba*I, and cloned between the *Sma*I and *Xba*I sites of the pBluescript II SK (+) plasmid.

To facilitate the nuclear localization of CrCre recombinase, *CrCRE* was fused in frame to *ble* from *Streptoalloteicus hindustanus*, conferring bleomycin/Zeocin resistance [47], to generate *ble*-*CrCRE* expression cassette I as follows. A 1.2-kb fragment containing the *HSP70-RBCS2* promoter fused to *ble* was amplified using phsp70_F3 and ble_R as the primers and the pMF59 plasmid [47] as a template. A 1.3-kb fragment containing *CrCRE* fused to the *RBCS2* terminator was amplified using Crcre_F2 and trbcS_R2 as primers and the pCrcre plasmid as a template. The 1.2- and 1.3-kb fragments were assembled into a single fragment by PCR with the phsp70_F3 and trbcS_R2 primers to form *ble-CrCRE* expression cassette I. The cassette DNA was purified using a PCR purification kit (Qiagen), digested with *Hind*III and *Xba*I, and cloned between the *Hind*III and *Xba*I sites of the pBluescript II SK (+) plasmid to generate the pbleCrcre plasmid (Fig 1). The pbleLCrcre plasmid harboring *ble-CrCRE* expression cassette II (Fig 1), in which a DNA sequence encoding a short artificial linker peptide, GGSGGR [48], was inserted in-frame between the 3'-end of *ble* and the 5'-end of *CrCRE*, was constructed as follows. First, PCR amplification was conducted using ble_R2 and Crcre_F3 as primers and the pbleCrcre plasmid as a template. Next, the amplified 5.5-kb fragment was circularized using an In-Fusion Cloning kit (Clontech) according to the manufacturer’s instructions.

### *C*. *reinhardtii* Strains and Growth Conditions

*C*. *reinhardtii* strains cc124 (mt−) and cc4350 (cw15 arg7-8 mt+, Chlamydomonas Resource Center) were used as recipients of the ploxP-aphVIII plasmid, whereas strain cc125 (mt+) was used in backcross experiments. Cells were cultivated mixotrophically at 25°C in Tris acetate phosphate (TAP) medium [49] supplemented with 10 μg ml^−1^ arginine if necessary under white fluorescent light (100 μmol photons m^−2^ s^−1^) with gentle shaking or on solid medium supplemented with 1.5% Bacto agar (BD Difco).

### Genetic Transformation of *C*. *reinhardtii* Strains

Nuclear transformation was performed using electroporation as described previously [50]. Briefly, the cells were grown for approximately 24 h until the cell densities reached 1 × 10^6^–2 × 10^6^ cells ml^−1^ in TAP medium. Cells were harvested by centrifugation at 800 × *g* for 5 min and washed with EP solution (30 mM HEPES, 5 mM MgSO_4_, 50 mM potassium acetate, 1 mM calcium acetate, 60 mM sucrose, pH 7.4), and suspend in EP solution to a final density of 1 × 10^8^–3 × 10^8^ cells ml^−1^. Then, 4 μl of 500 μg ml^−1^ DNA were added to 121 μl of the cell suspension. The cell suspension was placed into an electroporation cuvette with a 2-mm gap (Bio-Rad) and incubated at 15°C for 2 min. An exponential electric pulse of 2000 V/cm was applied to the suspension of strain cc124 using a GenePulser XCell™ (Bio-Rad) electroporation apparatus. The capacitance was set at 25 μF, and no shunt resistor was used. For strain cc4350, an exponential electric pulse of 700 V/cm at a capacitance of 600 μF was applied. After electroporation, cells were incubated at 15°C for 1 h and transferred to 10 ml of fresh TAP medium containing 40 mM sucrose. After incubation for 18 h at 25°C under dim light, the cells were collected by centrifugation at 800 × *g* for 5 min and selected on TAP agar plates supplemented with 20 μg ml^−1^ paromomycin (Wako) or 10 μg ml^−1^ Zeocin (Invitrogen). Each single colony developed on the agar plates was screened by PCR to identify gene-positive clones as described previously [51, 52] with some modifications. Each paromomycin-resistant (Pm^r^) clone was suspended in 10 μl of distilled water, into which the same volume of ethanol and 100 μl of 50% Chelex-100 (Bio-Rad, USA) were added. After incubation at 100°C for 10 min, cell debris was removed by centrifugation at 6000 rpm for 10 min. PCR was then performed using the supernatant as a template and aphVIII_F and aphVIII_R as primers to detect a partial *aphVIII* sequence or loxP_F and loxP_R as primers to detect the *loxP-P-aphVIII-T-loxP* sequence. In addition, PCR was performed to detect a partial *CrCRE* sequence using the primers Crcre_F4 and Crcre_R2, whereas detection of the full-length sequence of the *CrCRE* expression cassette, *ble-CrCRE* expression cassette I, or *ble-CrCRE* expression cassette II on the pCrcre, pbleCrcre, or pbleLCrcre plasmid was performed by two PCR amplifications using two primer sets: phsp70_F2 plus Crcre_R2 and Crcre_F4 plus trbcS_R2. The sequences of the primers used for the detection of transgenes are listed in Table 1.

### Southern Blot Analysis to Detect *aphVIII* Insertions

*C*. *reinhardtii* genomic DNA was extracted using a standard phenol-chloroform protocol [53]. Five micrograms of genomic DNA were digested with *Bam*HI, separated on 0.8% (w/v) agarose gel, and blotted onto a Hybond-N+ membrane (GE Healthcare, UK) by a standard capillary transfer method using 20 × SSC as a transfer buffer. The blotted membrane was then baked at 80°C for 2 h. An *aphVIII* fragment prepared by PCR using aphVIII_F and aphVIII_R as primers and the pSI103 plasmid as a template was labeled using a DIG High Prime DNA labeling and detection kit (Roche Applied Science). Hybridization and signal detection were performed according to the manufacturer’s instructions.

### Isolation of the Flanking Region of *loxP-P-aphVIII-T-loxP* Insertions

DNA regions flanking the *loxP-P-aphVIII-T-loxP* insertion were determined using inverse PCR as follows. Genomic DNA (0.5 μg) of transformants carrying a single copy of the *loxP-P-aphVIII-T-loxP* sequence was digested with *Bam*HI or *Pvu*II (Takara), both enzymes being single cutters of the ploxP-aphVIII plasmid (Fig 1). After inactivation of the restriction enzymes using phenol, digested DNA was ethanol-precipitated and dissolved in TE buffer. To amplify the 5′-flanking region of the *loxP-P-aphVIII-T-loxP* insertion, *Pvu*II-digested DNA was self-ligated and used as a template for an inverse PCR using trbcS_inv_F and phsp70_inv_R as primers. Similarly, to amplify the 3′-flanking region of the *loxP-P-aphVIII-T-loxP* insertion, *Bam*HI-digested DNA was self-ligated and used as a template for an inverse PCR using trbcS_inv_F and trbcS_inv_R as primers. The PCR reactions were conducted using Advantage-GC Genomic PCR mix (Clontech) using the step-down PCR protocol according to the manufacturer’s instruction. The resulting amplified fragments were purified using a QIAquick Gel Extraction kit (Qiagen) and cloned into the pGEMT-Easy plasmid (Promega). The nucleotide sequences of the fragments were then determined using dideoxy chain termination via a commercial service provided by Macrogen Japan Corp. The nucleotide sequences thus obtained were compared with the genome sequence of *Chlamydomonas* at a Joint Genome Institute site (https://phytozome.jgi.doe.gov/pz/portal.html#!info?alias=Org_Creinhardtii).

To verify excision of the *loxP-P-aphVIII-T-loxP* sequence integrated in the genomes of *C*. *reinhardtii* strains cc124_LPm1 and cc4350_LPm19 by Cre/*loxP*-mediated recombination, the insertion/excision regions were PCR-amplified using the primers designed from the sequences outside the *loxP-P-aphVIII-T-loxP* cassette sequence, namely primers LPm1_F and LPm1_R for the derivatives of strain cc124_LPm1 and primers LPm19_F and LPm19_R for the derivative of strain cc4350_LPm19. The nucleotide sequences of the PCR-amplified fragments were then determined as described previously.

### Reverse Transcription (RT)-PCR for the Detection of *CrCRE* Expression

Total RNA was extracted from cells grown in TAP medium to an OD_750_ of 2.0 using a TRIzol® plus RNA purification kit (Ambion), and the remaining DNA was digested using a TURBO DNA-free kit (Ambion) according to the manufacturer’s instructions. First-strand cDNA was synthesized using a PrimeScript™ RT reagent kit with gDNA Eraser (Perfect Real Time, TaKaRa) and an RT primer mix containing oligo (dT)18 and random hexamers. PCR to confirm the expression of *CrCRE* was performed using primers Crcre_F4 and Crcre_R2.

### Backcrossing and Segregation Analysis

Strain BLCP30, a derivative of cc124_LPm1 containing a single copy of *loxP* after Cre-mediated excision of the *loxP-P-aphVIII-T-loxP* sequence, was backcrossed to cc125 (mt+) to remove the *CrCRE* expression cassette. Mating was performed as described previously [54]. The resulting Zeocin-sensitive progenies were tested for the presence of the *loxP* sequence and the absence of the *CrCRE* expression cassette by PCR using primer sets LPm1_F2/LPm1_R4 and Crcre_F4/Crcre_R2.

### Accession Numbers

Sequence data from this study can be found in the DDBJ/NCBI data libraries under the accession numbers LC150884 (pCrcre), LC150885 (pbleCrcre), and LC150883 (pbleLCrcre).

## Results and Discussion

### Construction and Characterization of *C*. *reinhardtii* Transformants Carrying a Single *loxP-P-aphVIII-T-loxP* Insertion

**Fig 2:**
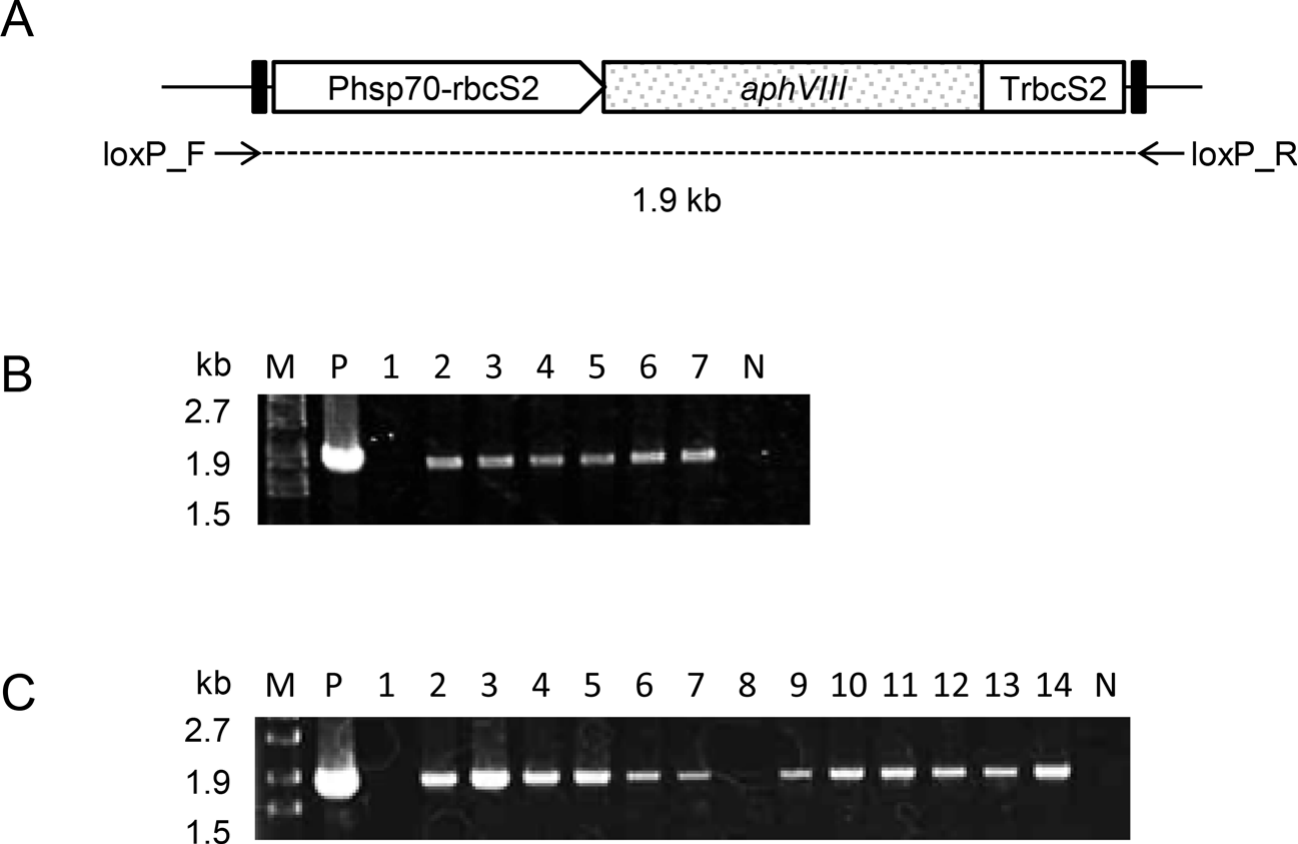
PCR analysis of transgenes in Pm^r^ transformants of strains cc124 and cc4350. (A) Map of the *aphVIII* expression cassette. The positions of two PCR primers that amplify the *loxP-P-aphVIII-T-loxP* sequence are shown below the map. (B) Agarose gel electrophoresis of the PCR-amplified *loxP-P-aphVIII-T-loxP* fragments. Lane M, DNA marker (λ-*Eco*T14 I digest) with molecular size in bp. The template DNAs were as follows: lane P, the ploxP-aphVIII plasmid; lanes 1–7, genomic DNAs of Pm^r^ transformants of strain cc124; lane N, no template. (C) Agarose gel electrophoresis of the PCR-amplified *loxP-P-aphVIII-T-loxP* fragments. Lane M, DNA marker (λ-*Eco*T14 I digest) with molecular size in bp. The template DNAs were as follows: lane P, the ploxP-aphVIII plasmid; lanes 1–14, genomic DNAs of Pm^r^ transformants of strain cc4350; lane N, no template.

**Fig 3:**
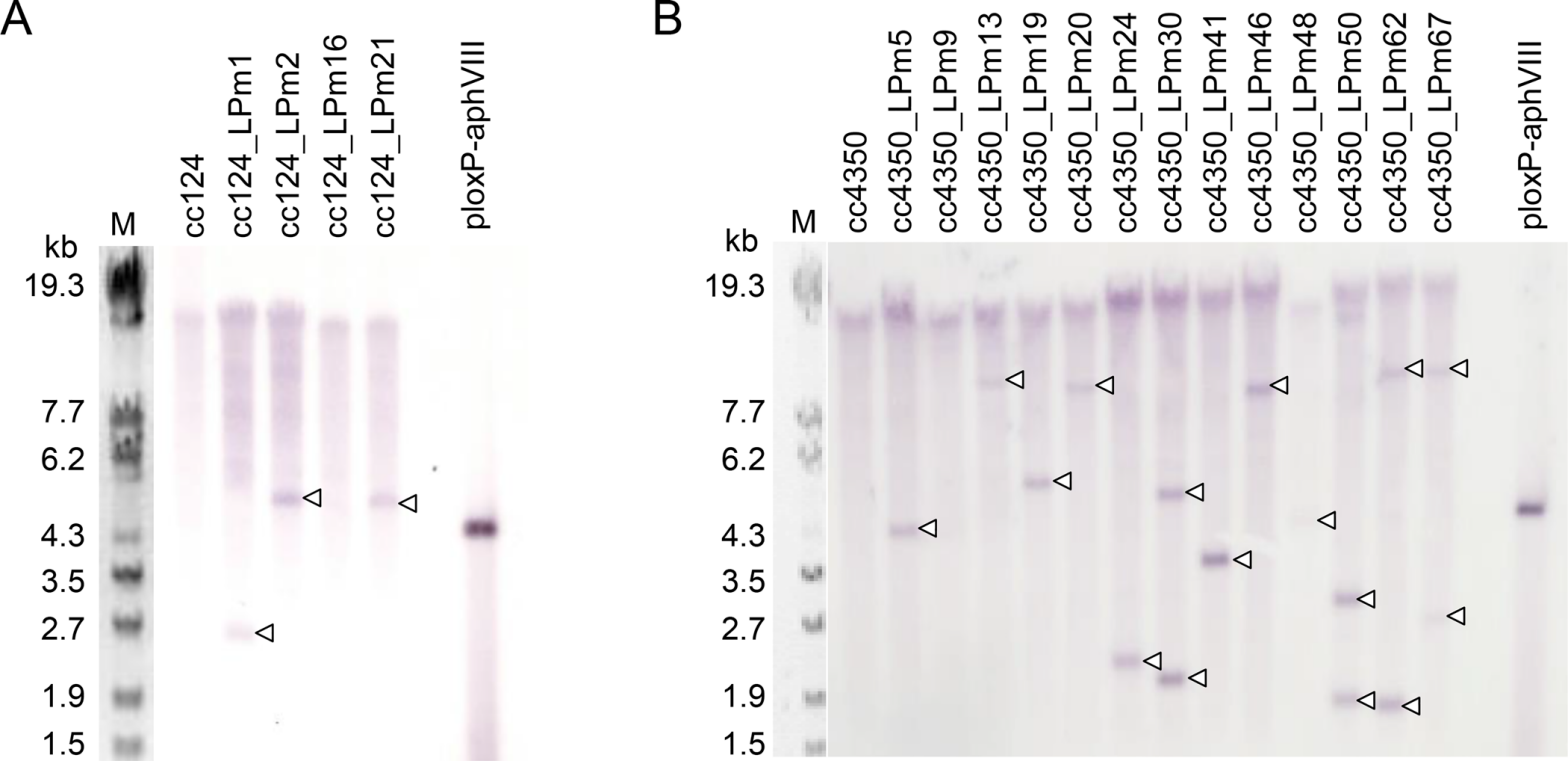
Analysis of the *aphVIII* copy number by Southern blotting. Genomic DNAs were isolated from Pm^r^ transformants of strains cc124 and cc4350, digested with *Bam*HI, and hybridized with a digoxigenin-labeled *aphVIII* fragment. (A) Southern blot analysis of the genomic DNAs of four Pm^r^ transformants derived from strain cc124. Lane M: DNA marker (λ-*Eco*T14 I digest) with molecular size in bp; next five lanes, *Bam*H1-digested genomic DNA of the strains indicated above the lanes; lane ploxP-aphVIII, the ploxP-aphVIII plasmid digested with *Bam*HI. (B) Southern blot analysis of the genomic DNAs of 13 Pm^r^ transformants derived from strain cc4350. Lane M, DNA marker (λ-*Eco*T14 I digest) with molecular size in bp; next 14 lanes, *Bam*HI-digested genomic DNAs of the strains indicated above the lanes; lane ploxP-aphVIII, the ploxP-aphVIII plasmid digested with *Bam*HI.

The *Eco*RI-linearized ploxP-aphVIII plasmid was introduced in strains cc124 and cc4350, and 16 and 79 Pm^r^ transformants, respectively, were isolated. The sequences of the *loxP*-flanked *aphVIII* expression cassettes (*loxP-P-aphVIII-T-loxP*) integrated in the genomes of these transformants were analyzed by PCR with primers loxP_F and loxP_R, and the integration of the whole *loxP-P-aphVIII-T-loxP* sequence was confirmed in 6 cc124-derived and 13 cc4350-derived Pm^r^ transformants (Fig 2). Southern blot analyses were performed to detect the *aphVIII* sequence in *Bam*HI-digested DNAs isolated from 4 cc124-derived and 13 cc4350-derived transformants carrying the whole *loxP-P-aphVIII-T-loxP* sequence. The *Bam*HI restriction endonuclease cuts ploxP-aphVIII plasmid once at the 3′-end of *aphVIII*; therefore, the number of bands revealed by the probe corresponds to the number of *aphVIII* insertions in the host genomes. The top band in each lane was thought to be non-specific signals as the band was also observed in the lanes for the wild type strains, cc124 and cc4350. The analyses thus revealed that most transformants contained a single *aphVIII* insertion, whereas the remainder carried two (Fig 3). The sizes of the majority of the bands were different from each other, indicating that most of the *loxP-P-aphVIII-T-loxP* insertions were located at different loci on the *C*. *reinhardtii* chromosomes. Two transformants, cc124_LPm1 and cc4350_LPm19, each carrying a single copy of the *loxP-P-aphVIII-T-loxP* insertion, were selected for further studies to demonstrate the excision of the *loxP-P-aphVIII-T-loxP* insertion by CrCre recombinase.

To map the insertion sites of the *loxP-P-aphVIII-T-loxP* sequence in strains cc124_LPm1 and cc4350_LPm19, flanking DNA regions were amplified using inverse PCR and sequenced as described in the Materials and Methods. The nucleotide sequences of the flanking regions were then aligned to the *C*. *reinhardtii* genome sequence [gene model version JGI 5.5 (Phytozome 10), Joint Genome Institute: http://www.phytozome.net/chlamy]. In strain cc124_LPm1, the *loxP-P-aphVIII-T-loxP* sequence was inserted in a gene of unknown function (Cre08.g362400, 1,099,596…1,102,295 on chromosome 8) at location 1,099,903, whereas the insertion site in the cc4350_LPm19 genome was mapped to multiple locations in the genome, which could not be determined unequivocally (Table 2).

**Table 2:** The mapped locations of genomic sequence flanking of the *loxP-P-aphVIII-T-loxP* sequence in the cc4350_LPm19 genome.

		Position		
		start	end	identity
5' flanking sequence	chromosome_2	8931710	8932113	399/404 (98.8)
	chromosome_3	6813235	6812832	404/404 (100)
	chromosome_3	6861657	6862060	403/404 (99.8)
	chromosome_4	803530	803127	397/404 (98.3)
	chromosome_4	1679437	1679840	404/404 (100)
	chromosome_4	1682491	1682894	402/404 (99.5)
	chromosome_4	2994518	2994921	404/404 (100)
	chromosome_9	5934421	5934824	404/404 (100)
	chromosome_13	4248888	4248485	404/404 (100)
	chromosome_14	2096415	2096818	404/404 (100)
	chromosome_15	1378457	1378054	404/404 (100)
	chromosome_17	804556	804959	404/404 (100)
	chromosome_17	816331	816734	404/404 (100)
	chromosome_17	2661431	2661028	404/404 (100)
	scaffold_22	160645	160242	401/404 (99.3)
3' flanking sequence	chromosome_2	8932114	8934462	2324/2349 (98.7)
	chromosome_3	6862061	6864404	2344/2344 (100)
	chromosome_3	6812831	6810512	2339/2344 (98.8)
	chromosome_4	803126	800786	2324/2344 (99.0)
	chromosome_4	1682895	1685238	2340/2344 (99.8)
	chromosome_4	2994922	2997265	2343/2344 (99.9)
	chromosome_8	2874233	2872350	1883/1884 (99.9)
	chromosome_9	5934825	5937168	2344/2344 (100)
	chromosome_13	4248484	4246141	2342/2344 (99.9)
	chromosome_14	2096819	2099151	2342/2344 (99.4)
	chromosome_15	1378053	1375720	2342/2344 (99.5)
	chromosome_17	804960	807292	2341/2344 (99.4)
	chromosome_17	816735	819078	2341/2344 (99.9)
	chromosome_17	2661027	2658684	2343/2344 (99.9)
	scaffold_22	160241	157991	2229/2251 (99.0)

### Demonstration of CrCre Recombinase-Mediated Site-Specific Recombination in *C*. *reinhardtii*

**Fig 4:**
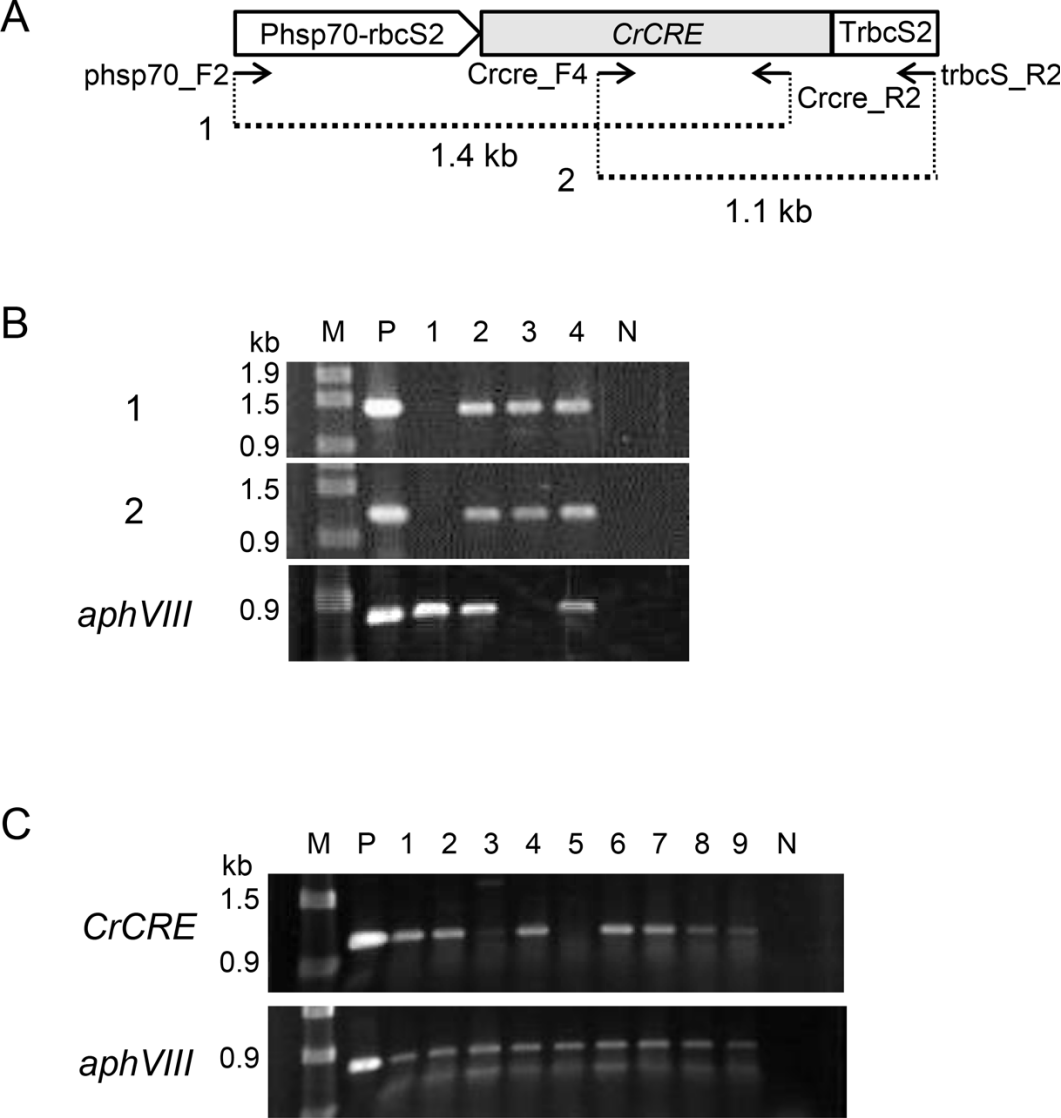
PCR amplification of the *CrCRE* expression cassette sequence integrated in the genomes of Zeo^R^ transformants. (A) The structure of the *CrCRE* expression cassette and the sizes of the PCR products (1 and 2) amplified using two different primer sets. (B) Agarose gel electrophoresis of three different PCR products. Panels 1 and 2, detection of PCR products 1 and 2; panel *aphVIII*, detection of *aphVIII*. Lane M, DNA marker (λ-*Eco*T14 I digest) with molecular size in bp. The template DNAs were as follows: lane P, the pCrcre plasmid for panels 1 and 2 and the ploxP-aphVIII plasmid for panel aphVIII; lanes 1–4, genomic DNAs of Zeo^r^ transformants of strain cc4350_LPm19; lane N, no template. For panels 1 and 2, the primer sets phsp70_F/Crcre_R2 and Crcre_F4/trbcS_R2, respectively, were used, whereas for panel *aphVIII*, the primer set aphVIII_F/aphVIII_R was used. (C) Reverse transcription-PCR analysis of the expression of *CrCRE* and *aphVIII* in the Zeo^R^ transformants of strain cc4350_LPm19 containing the *CrCRE* expression cassette. Panel *CrCRE*, detection of *CrCRE* transcripts. Panel *aphVIII*, the detection of *aphVIII* transcripts. Lane M, DNA markers (λ-*Eco*T14 I digest) with molecular size in bp. Lane P, template DNAs were extracted from the pCrcre plasmid for *CrCRE* detection and the ploxP-aphVIII plasmid for *aphVIII* detection; lanes 1–9, RNAs were isolated from the Zeo^R^ transformants of strain cc4350_LPm19 containing the *CrCRE* expression cassette; lane N, minus reverse transcriptase. The same lane numbers within Fig4(A) and 4(B) do not represent the same transformants.

To examine excision of the *loxP-P-aphVIII-T-loxP* sequence by CrCre recombinase, the pCrcre plasmid carrying the *CrCRE* expression cassette was introduced into strain cc4350_LPm19 via co-transformation with the pMF59 plasmid carrying *ble* conferring Zeocin resistance (Zeo^r^), and Zeo^r^ transformants were screened on TAP agar plates containing Zeocin. The existence of the *CrCRE* expression cassette in 226 Zeo^r^ transformants was then examined by PCR with two primer sets: phsp70_F2 plus Crcre_R2 and Crcre_F4 plus trbcS_R2 (Fig 4A). The entire *CrCRE* cassette sequence was detected in 46 Zeo^r^ transformants. We first expected that all transformants carrying the intact *CrCRE* expression cassette would be Pm-sensitive (Pm^s^), as the *loxP-P-aphVIII-T-loxP* sequence might have been excised by CrCre recombinase. However, only 1 of the 46 transformants was Pm^s^, and excision of the *aphVIII* sequence in the Pm^s^ transformant was confirmed by PCR (Fig 4B).

This unexpectedly low excision rate of the *loxP-P-aphVIII-T-loxP* sequence in the pCrcre transformants may be due to several reasons. The first possibility was the low expression of *CrCRE* from the *CrCRE* expression cassette. Then, *CrCRE* expression was examined in nine randomly selected pCrcre transformants by RT-PCR using PCR primers Crcre_F4 and Crcre_R2. *CrCRE* expression was detected in seven of nine strains, whereas *aphVIII* expression was detected in all strains (Fig 4C).

To overcome potential problems including malfunction of translation and/or inefficient nuclear translocation of the CrCre protein, *CrCRE* was fused to *ble* to construct the pbleCrcre plasmid (Fig 1). There were two reasons for the construction of the Ble-CrCre fusion proteins: (i) As the level of resistance to Zeocin is proportional to the protein expression level of Ble [55], transformants expressing the Ble-CrCre fusion protein at high levels could readily be isolated by selecting for Zeo^r^ at higher levels. (ii) Ble is a bleomycin-binding protein that localizes in the nucleus [47]; thus, fusion with the Ble protein would further facilitate the nuclear translocation of the CrCre recombinase.

**Fig 5:**
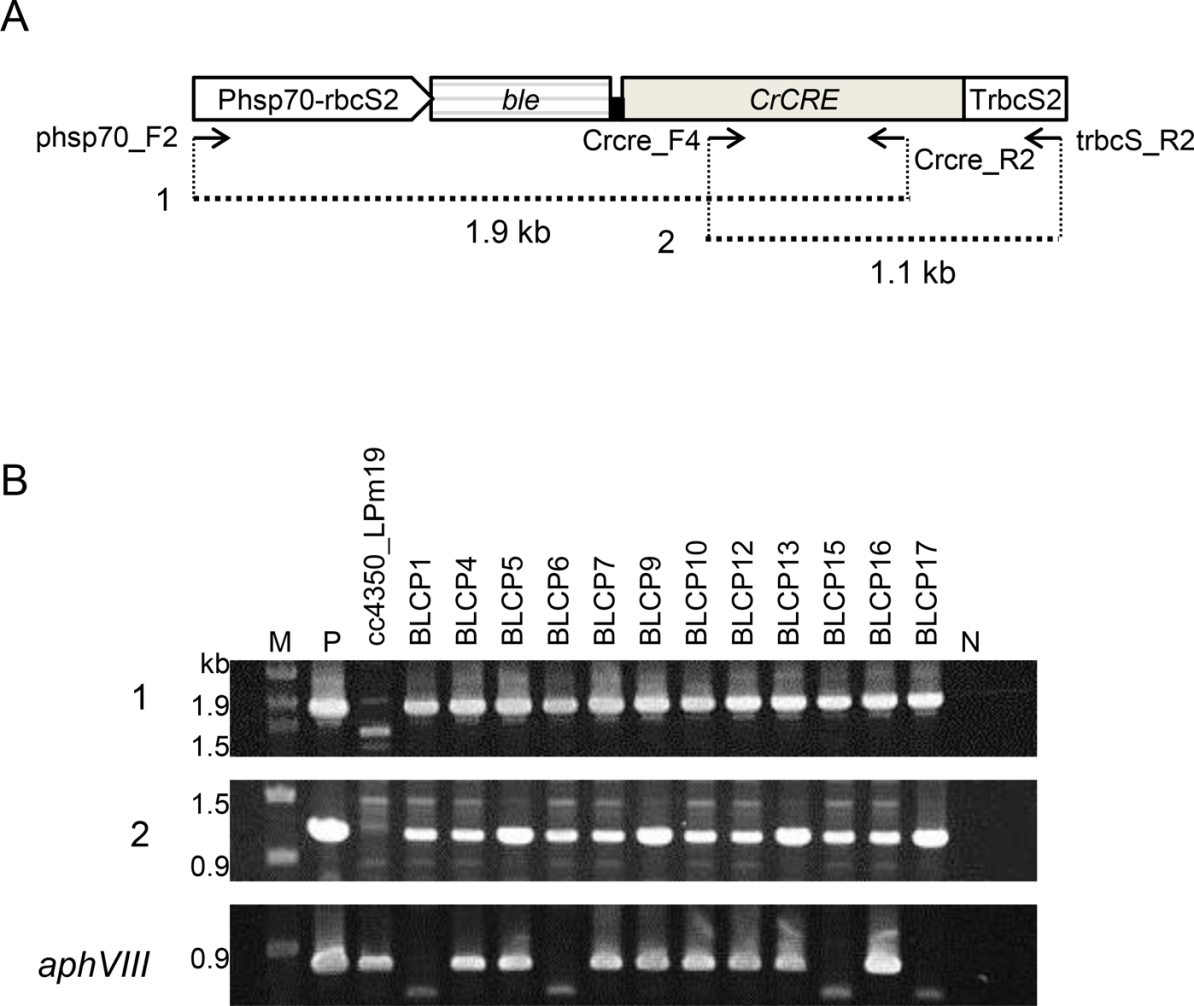
PCR amplification of the *ble-CrCRE* expression cassette II sequence integrated into the Zeo^r^ transformants. (A) The structure of *ble-CrCRE* expression cassette II, positions of two PCR primer sets, and PCR products 1 and 2 amplified with the two primer sets are shown. (B) Agarose gel electrophoresis of three different PCR products. Panels 1 and 2, detection of PCR products 1 and 2; panel *aphVIII*, detection of *aphVIII*. Lane M, DNA marker (λ-*Eco*T14 I digest) with molecular size in bp. The template DNAs were as follows: lane P, the pbleLCrcre plasmid for panels 1 and 2 and the ploxP-aphVIII plasmid for panel *aphVIII*; next 13 lanes, genomic DNAs of the strains indicated above the lanes; lane N, no template. The Zeo^r^ transformants of strain cc4350_LPm19 carrying *ble-CrCRE* expression cassette II were named BLPC. The *aphVIII* sequence was not detected in strains BLCP1, BLCP6, BLCP15, and BLCP17.

When the pbleCrcre plasmid carrying *ble-CrCRE* expression cassette I was introduced into strain cc4350_LPm19 by selecting Zeo^r^ transformants, excision of the *loxP-P-aphVIII-T-loxP* sequence was not detected. We expect that the CrCre recombinase directly fused to the Ble protein was not functional in the Zeo^r^ transformants probably because two domains in the bifunctional fusion protein were not effectively separated each other [56, 57], or that the fusion protein had a high chance of misfolding [58]. A fusion gene encoding the Ble protein fused to CrCre recombinase via a flexible linker of six amino acids was then designed. The pbleLCrcre plasmid harboring *ble-CrCRE* expression cassette II ([the *HSP-RBCS* promoter]–[the *ble*-linker-*CrCRE* fusion protein gene]–[the *RBCS* terminator]) (Fig 1) was introduced into strain cc4350_LPm19, and Zeo^r^ transformants were screened on TAP agar plates containing Zeocin. Seventy-four Zeo^r^ transformants were obtained, and the existence of the *ble-CrCRE* expression cassette II sequence in the transformants was examined by PCR as described previously (Fig 5A). In the genomes of 12 of 74 transformants, the intact *ble-CrCRE* expression cassette II was integrated (Fig 5B). The absence of the *aphVIII* sequence in the genome of 12 transformants was next examined by PCR using primers aphVIII-F and aphVIII-R. The *aphVIII* sequence was not detected in four of the transformants (Fig 5B). These four *aphVIII-*free transformants were Pm^s^, whereas the remaining eight transformants were Pm^r^. The four *aphVIII*-free transformants were named strains BLCP1, BLCP6, BLCP15, and BLCP17. From the remaining eight Pm^r^ transformants, *aphVIII*-free descendants were isolated after single-colony isolation repeated 2–6 times, indicating that CrCre recombinase-mediated site-specific recombination could be delayed, requiring many generations to elapse before recombination.

**Fig 6:**
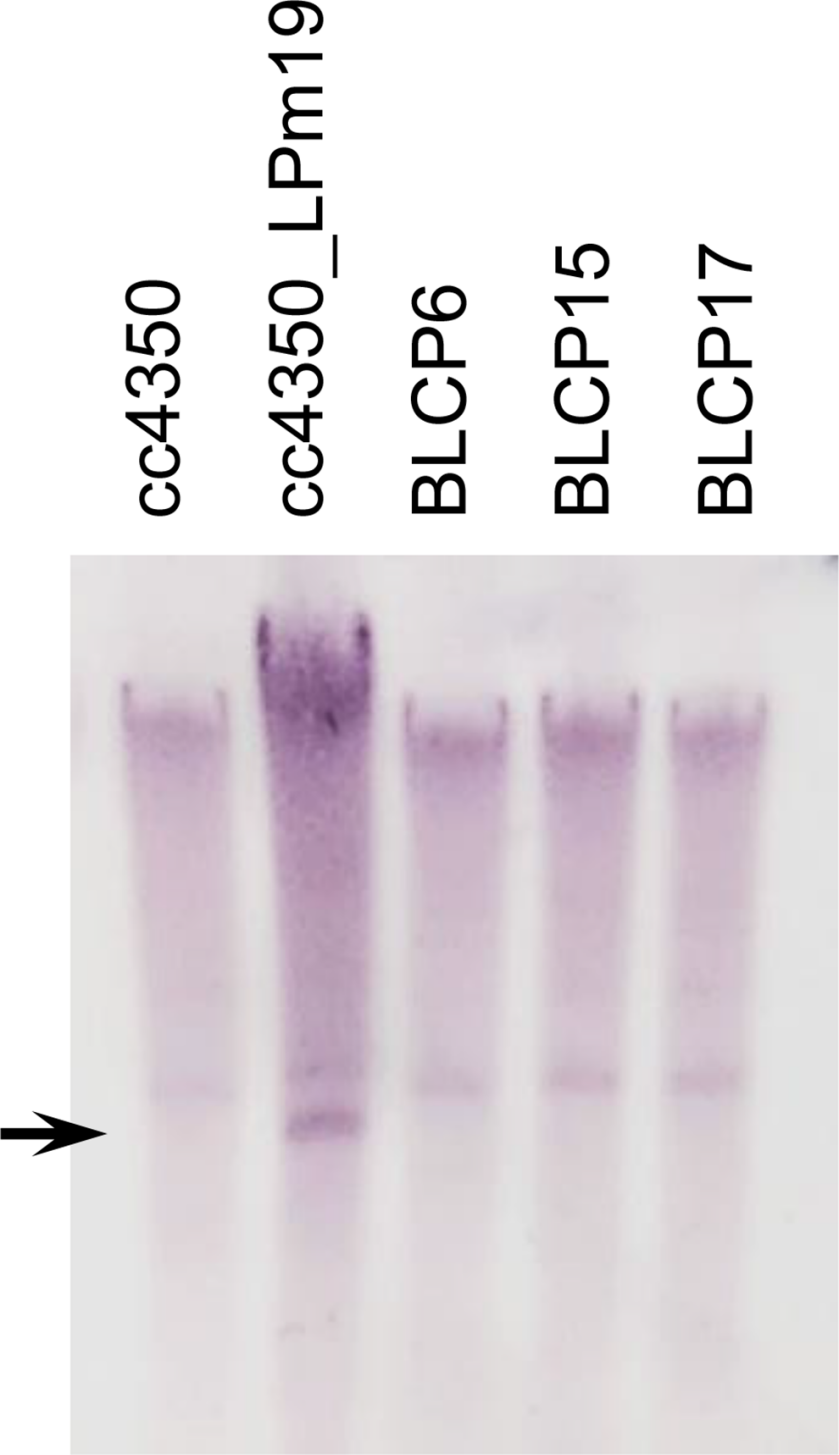
Southern blot analysis of transgenes in strains BLCP6, BLCP15, and BLCP17. All DNAs were digested with *Bam*HI, electrophoresed, and hybridized with a digoxigenin-labeled *aphVIII* fragment. The arrow indicates the restriction fragment containing the *loxP-P-aphVIII-T-loxP* sequence in the genome of strain cc4350_LPm19.

**Fig 7:**
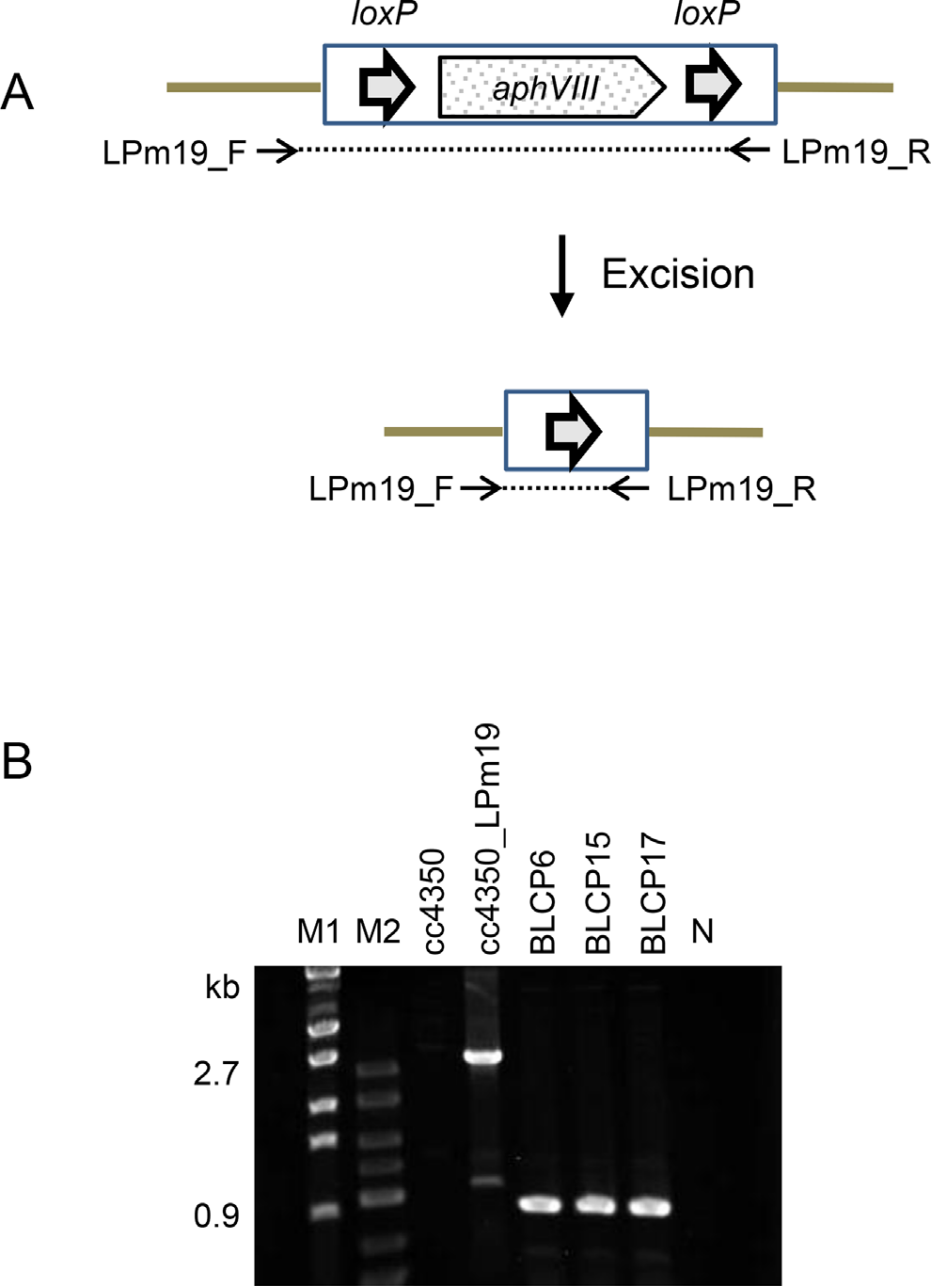
PCR analysis to detect Cre-mediated excision of the *loxP-P-aphVIII-T-loxP* sequence. (A) The box indicates a partial sequence of the ploxP-aphVIII plasmid, and solid lines indicate the genomic sequence. The *loxP* sites are indicated by short arrows. The two arrows beneath the image denote the PCR primer set used for the amplification of either a 2.7-kb fragment from DNA without the excision event or a 0.9-kb fragment from DNA with the Cre-mediated excision. (B) Agarose gel electrophoresis of the 2.7- and 0.9-kb fragments. Lane M1, DNA size marker (λ-*Eco*T14 I digest) with molecular size in bp; lane M2, DNA size marker (50–2500 bp, Lonza); next five lanes, PCR fragments amplified from genomic DNAs of the strains indicated above the lanes; lane N, no template control.

Genomic DNA was extracted from strains BLCP6, BLCP15, and BLCP17, and Southern blotting with a probe specific for the *aphVIII* sequence was performed (Fig 6). The *aphVIII* signal was not detected in these three strains. The DNA sequences of strains BLCP6, BLCP15, and BLCP17 corresponding to the *loxP-P-aphVIII-T-loxP* integration site in their parental strain, cc4350_LPm19, were analyzed by PCR using primers LPm19_F and LPm19_R. A 2.7-kb fragment was amplified from strain cc4350_LPm19, whereas a 0.9-kb fragment was amplified from BLCP6, BLCP15, and BLCP17 (Fig 7). The nucleotide sequence of the 0.9-kb fragment revealed that the *loxP-P-aphVIII-T-loxP* sequence was accurately excised by Cre/*loxP*-mediated recombination, leaving a single copy of *loxP*.

**Fig 8:**
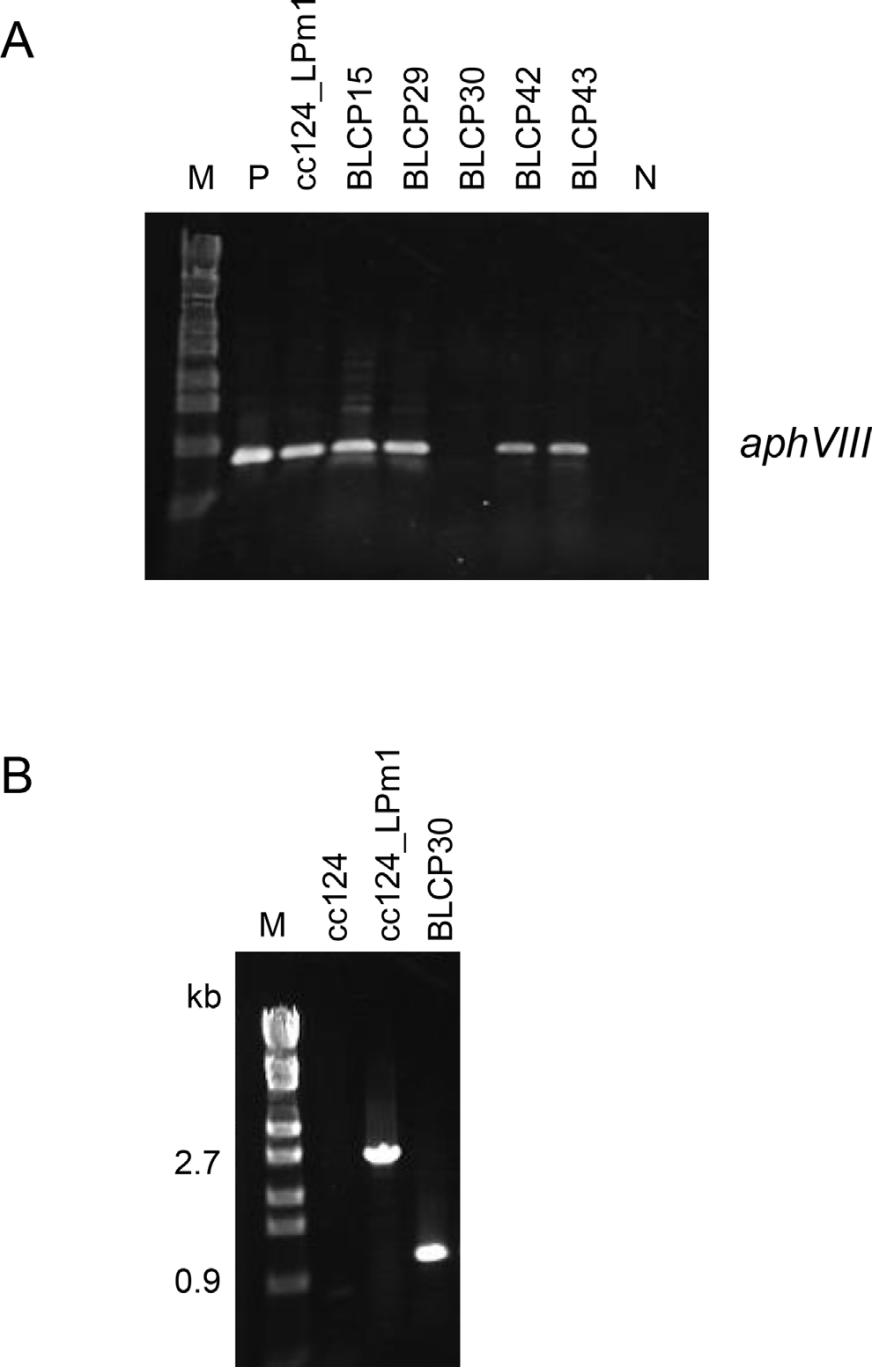
PCR analysis to detect the Cre-mediated excision of the *loxP-P-aphVIII-T-loxP* sequence. (A) Agarose gel electrophoresis to detect *aphVIII*. Lane M, DNA size marker (λ-*Eco*T14 I digest) with molecular size in kb. The template DNAs were as follows: lane P, the ploxP-aphVIII plasmid; next five lanes, DNAs of the strains indicated above the lanes; lane N, no template. The Zeo^r^ transformants of strain cc124_LPm1 carrying *ble-CrCRE* expression cassette II were named BLCP. No *aphVIII* signal was detected in strain BLPC30 indicating the excision of the *loxP-P-aphVIII-T-loxP* sequence in this strain. (B) PCR amplification of the *loxP-P-aphVIII-T-loxP* integration sites of strains cc124, cc124_LPm1, and BLCP30.

*ble-CrCRE* expression cassette II was also introduced into strain cc124_LPm1. In the genomes of 5 of the 163 Zeo^r^ transformants, the entire cassette was integrated, and one of the five Zeo^r^ transformants was Pm^s^. This strain was named BLCP30, and the *aphVIII* sequence was absent in its genome (Fig 8A). The DNA sequence of strain BLCP30 corresponding to the *loxP-P-aphVIII-T-loxP* insertion site in its parental strain, cc124_LPm1, was analyzed by PCR using primers LPm1_F2 and LPm1_R4. The 2.9-kb fragment was amplified from strain cc124_LPm1, whereas a 1.1-kb fragment was amplified from strain BLCP30 (Fig 8B). The accurate excision of *loxP-P-aphVIII-T-loxP* leaving a single copy of *loxP* was confirmed by nucleotide sequencing of the 1.1-kb fragment.

### Removal of *ble-CrCRE* Expression Cassette II via Backcross to a Wild-Type Strain

**Fig 9:**
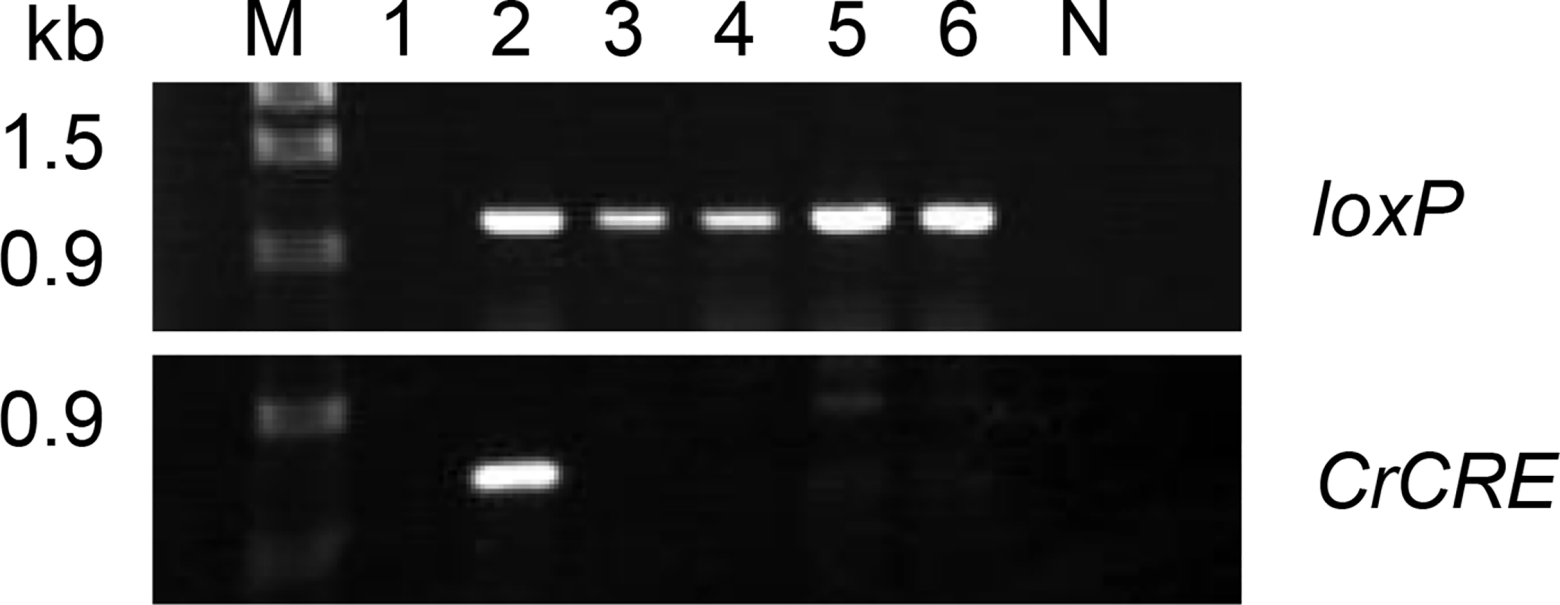
Removal of *ble-CrCRE* expression cassette II sequence by backcross. Agarose gel electrophoresis of PCR-amplified *loxP* and *CrCRE* sequences from the genomes of four progeny obtained by backcross between strains BLCP30 and cc125. Lane M, DNA size marker (λ-*Eco*T14 I digest) with molecular size in kb. Template DNAs were extracted from the following: lane 1, strain cc124; lane 2, strain BLCP30; lanes 3–6, progeny; lane N, no template.

*ble-CrCRE* expression cassette II remained in the genomes of *aphVIII*-cured derivatives. The presence of *ble-CrCRE* expression cassette II in a host genome hinders the subsequent introduction of a *loxP*-flanked marker gene into the host. Furthermore, constitutive expression of CrCre recombinase may induce DNA damage at off-target sites [59, 60, 61, 62]. To remove *ble-CrCRE* expression cassette II from strain BLCP30, which is a descendent of strain cc124 (mt−), this strain was crossed to the wild-type strain cc125 (mt+). Seven tetrads were dissected, and 25 recombinant progenies were isolated. They were grown on TAP plates for 72 h and tested for their phenotypes. In total, 12 of 25 progeny were sensitive to Zeocin, and four carried the *loxP* sequence. The absence of *CrCRE* in the genomes of the four progeny was also confirmed by PCR (Fig 9).

## Conclusion

**Fig 10:**
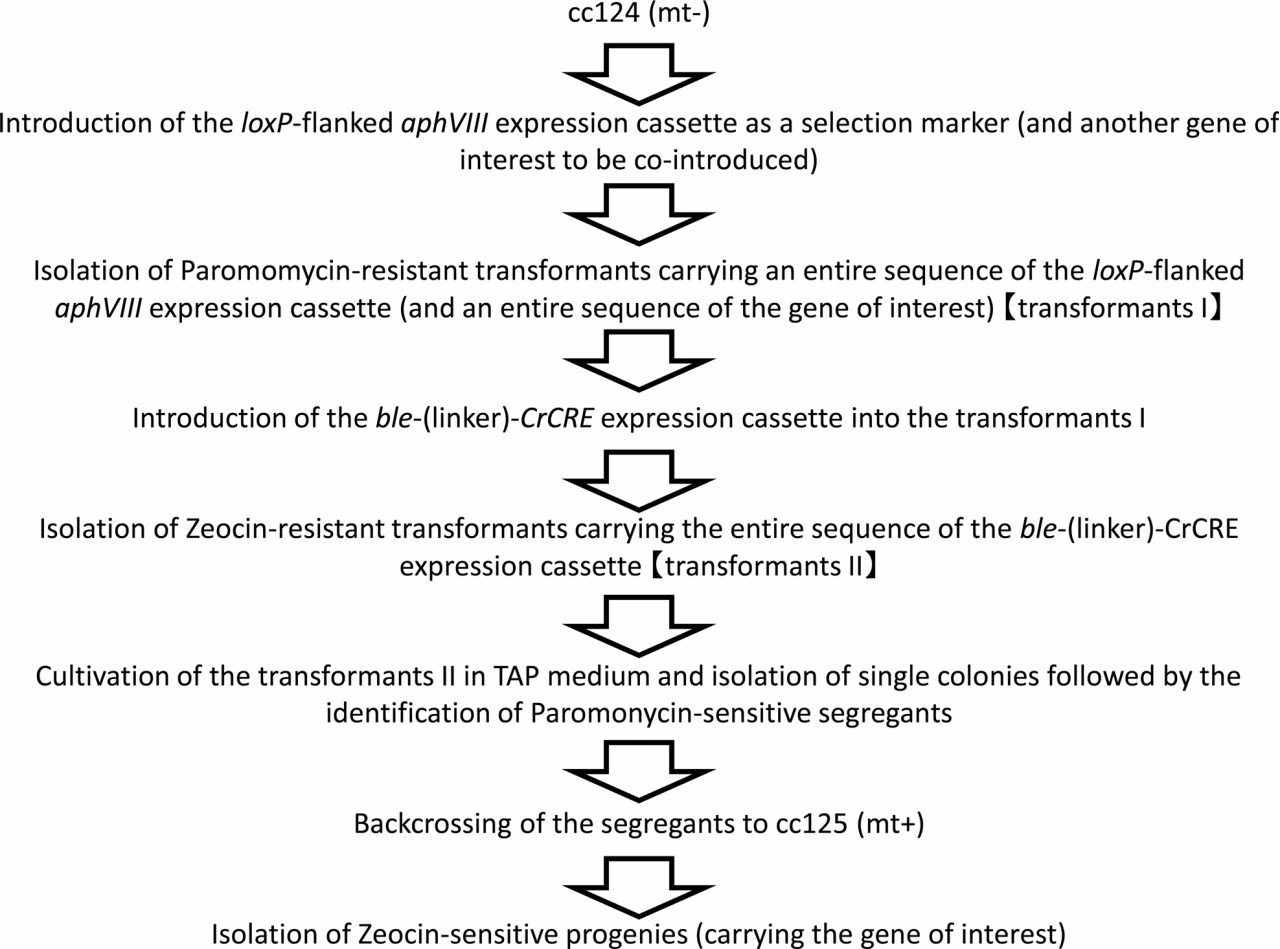
Summary of the method to obtain marker-free transgenic strains in *C*. *reinhardtii*.

In this study, we developed a method to obtain marker-free transgenic strains in *C*. *reinhardtii*, and the steps of the method are outlined in Fig 10. The Cre/*loxP*-mediated precise marker excision method applicable to transgenic *C*. *reinhardtii* could further increase the potential of this organism for use in basic and applied research.
